# Grassland Carbon Budget and Its Driving Factors of the Subtropical and Tropical Monsoon Region in China During 1961 to 2013

**DOI:** 10.1038/s41598-017-15296-7

**Published:** 2017-11-07

**Authors:** Li Zhang, GuangSheng Zhou, YuHe Ji, YongFei Bai

**Affiliations:** 1 State Key Laboratory of Vegetation and Environmental Change, Institute of Botany, Chinese Academy of Sciences, Beijing, 100093 China; 2 University of Chinese Academy of Sciences, Beijing, 100049 China; 3 Chinese Academy of Meteorological Sciences, Beijing, 100081 China

## Abstract

The southern grasslands are an integral part of the grassland ecosystems of China and play an essential role in the terrestrial carbon cycle of the country. We reproduced the spatiotemporal dynamics of the carbon budget of southern grasslands from 1961 to 2013 using the Terrestrial Ecosystem Model and our results showed that the annual carbon budget varied from −8.12 to 6.16 Tg C y^−1^ with an annual average of 0.45 Tg C y^−1^ during the study period. Overall, southern grasslands acted as a weak carbon sink and sequestrated 23.83 Tg C from 1961 to 2013. At the seasonal scale, southern grasslands acted as a carbon sink in wet seasons but as a carbon source in dry seasons. During the study period, temperature and precipitation were the main factors driving carbon budget dynamics at the seasonal scale, while soil moisture was the main driving factor at the annual scale. Over the entire study region, 71.81% of the area switched to being a carbon sink while only 5.90% remained stable and the strong carbon sinks were mainly found in the southern, northern and western areas of the southern grasslands.

## Introduction

Grasslands are one of the most extensive terrestrial ecosystem on Earth, and they play a crucial role in the circulation of energy and matter, global climate change and the global carbon balance^1,2^. Based on a national grassland resource survey^3^, the grasslands of China cover approximately 3.95 × 10^6^ km^2^, which is approximately 41.1% of the total territory of the country. It has been reported that grasslands account for 20% of the total carbon in both the soil and vegetation on Earth^4^, and the relationship between the global carbon cycle and the carbon budgets of grasslands has become one of the most important questions in ecology and environmental science^5–7^.

Many previous studies have estimated the productivity and carbon storage of grassland ecosystems^8,9^, including the annual NPP over different periods^10–12^ and for different types of grasslands^13–16^. Most studies on the carbon pools or carbon budgets of grasslands have mainly focused on regions with continuous areas of vegetation cover, such as the grasslands of the Qinghai-Tibetan Plateau^17–20^ and Inner Mongolian^21–24^. Several studies have investigated the total carbon budget for all the grassland ecosystems in China^14,25–28^, and different studies^2,13,14,27,29–32^ have found different carbon pool and carbon flux estimates. The vegetation carbon storage and density of the grasslands of China have been found to range from 0.56~4.67 Pg C and 215.80~1148.20 g C m^−2^, respectively, and the soil carbon storage and density^2,14,33^ have been found to range from 16.70~41.00 Pg C and 10.00~15.10 kg C m^−2^, respectively. Consequently, the quantitative assessment of carbon exchange rates and the accurate simulation of the spatiotemporal variation in carbon fluxes are crucial for forecasting trends under future climate change and for understanding the mechanisms that control global change^34–36^.

Southern China is located in the subtropical and tropical monsoon region and experiences distinct seasons, and the grasslands in this zone have a disjunct distribution, are small and minimally affected by human activities. However, although there have been several studies on the carbon budget of southern grasslands^37–45^, there has been relatively little research on the spatiotemporal dynamics of the regional carbon budget of southern China and its control mechanisms since 1961, which limits our understanding of the response of the carbon budget dynamics of southern grassland ecosystems to climate change.

In this work, we calibrated and verified the Terrestrial Ecosystem model using information about nitrogen and carbon fluxes and pools acquired from field observation and the literature. We then simulated and analyzed the spatiotemporal dynamics of the carbon budget and its control mechanisms in southern grasslands from 1961 to 2013.

## Results

### Model Verification

**Figure 1:**
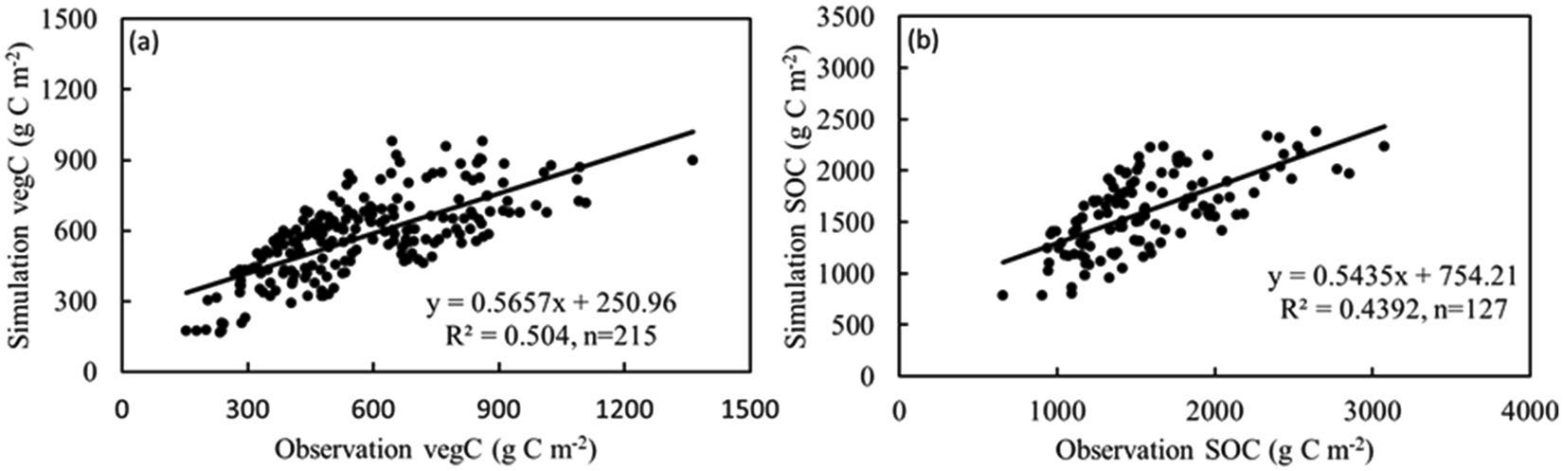
Comparisons of simulated vegetation (**a**) and soil organic carbon densities (**b**) with the observation data of 2011~2013.

We verified the TEM results with the observed vegetation and soil organic carbon density data sets from 2011 to 2013 (Fig. 1). First, we removed unreasonable values from the observation data, such as above- or below-ground vegetation densities or soil carbon densities in all soil layers equal to zero, as well as the data for parameter calibration, and we then used the Pauta criterion (3δ method) to exclude the observation data that were more than 3-fold greater than of the standard deviation of the observation data and their average. Finally, we used the remaining observation data to verify the TEM simulation results. The parameterized TEM-simulated vegetation carbon (R^2^ = 0.50, n = 215) and soil organic carbon (R^2^ = 0.44, n = 127) data matched the observation data well, exceeding the confidence level of 99%. The average measured vegetation and soil organic carbon density values were 572.70 g C m^−2^ and 1564.60 g C m^−2^, respectively, which were close to the corresponding simulated values of 574.94 g C m^−2^ and 1604.56 g C m^−2^.

### Temporal Dynamics of Precipitation and Temperature

**Figure 2:**
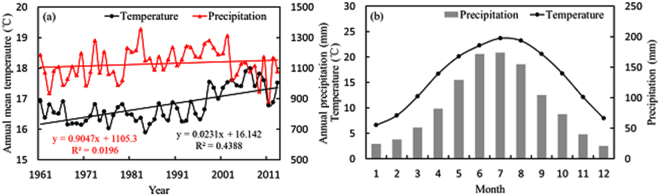
Inter-annual (**a**) and monthly (**b**) variations of temperature and precipitation during 1961 to 2013.

The average annual temperature in southern grasslands increased remarkably (R^2^ = 0.44, P < 0.001, N = 53; Fig. 2a) during 1961 to 2013, and the annual precipitation varied greatly from year to year, with an annual average of 1057.35 mm (Fig. 2a). The study region was seriously affected by the Asian Monsoon and experienced considerable seasonal changes in precipitation with obvious wet (April to September) and dry seasons in one year (Fig. 2b). During wet seasons, the total precipitation accounted for 77.16% of the annual precipitation, on average, from 1961 to 2013. The mean temperature was 21.10 °C during wet seasons, which was approximately twice that in dry seasons (10.71 °C). Therefore, the climate was wetter and warmer during wet seasons, especially during the summer season of June to August.

### Temporal Dynamics of the Carbon Budget

**Figure 3:**
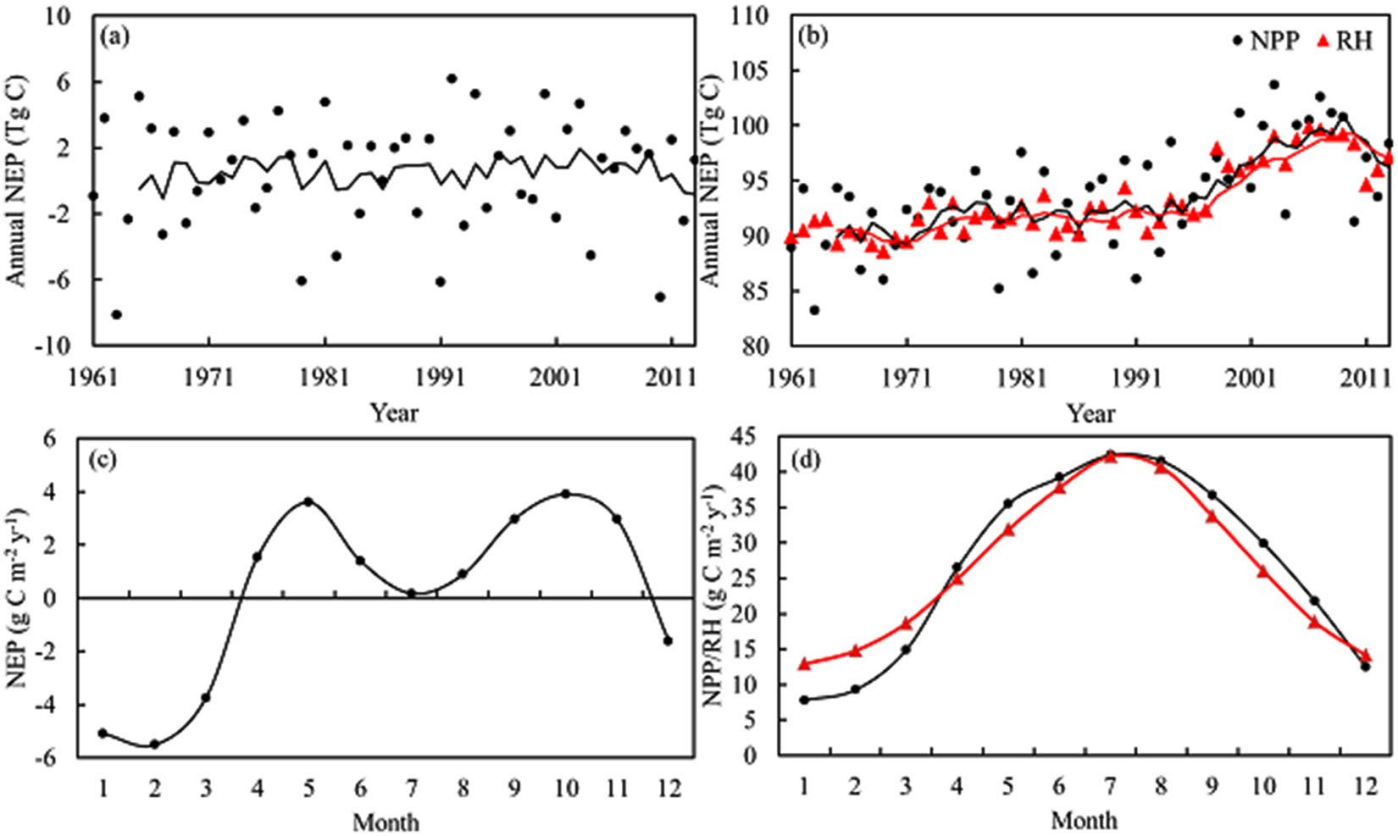
Inter-annual and monthly variations of carbon budget during 1961 to 2013.

In the TEM, net ecosystem production (NEP) was defined as the difference between net primary production (NPP) and heterotrophic respiration (RH). The annual NEP in southern grasslands ranged from −8.12 to 6.16 Tg C y^−1^ from 1961 to 2013, with an annual average of 0.45 Tg C y^−1^ (Fig. 3a). Thus, the entire study region (0.29 × 10^6^ km^2^) acted as a weak carbon sink and fixed 23.83 Tg C during the study period with large inter-annual variations. Compared with NEP, the annual NPP and RH showed different temporal dynamics (Fig. 3b); both showed significantly increasing trends from 1961 to 2013 (R^2^ = 0.35, P < 0.001, N = 53 and R^2^ = 0.68, P < 0.001, N = 53, respectively). The rate of increase of NPP (0.18 Tg C y^−1^) was greater than that of RH (0.17 Tg C y^−1^), and the difference between these rates was the basis for the annual fluctuations in NEP in southern grasslands from 1961 to 2013. The average annual NPP was 93.63 Tg C y^−1^ during 1961 to 2013, and the annual RH ranged from 89.9 to 97.1 Tg C with an annual average of 93.2 Tg C y^−1^.

The seasonal variations in monthly NPP and RH exhibited the same pattern, decreasing from July to December and increasing from January to July (Fig. 3d), and the differences in the monthly NPP and RH led to the seasonal variation in NEP. The seasonal variation in monthly NEP showed a distinct bimodal pattern with the maximum appearing in May and October (Fig. 3c). The NEP in summer was close to zero because almost all the carbon fixed by grasslands from the atmosphere was released by the soil under high temperature and high moisture conditions. Therefore, the southern grasslands acted as a carbon sink in wet seasons and as a carbon source in dry seasons.

### Response of NEP to Climate Variables

Climatic variables, such as precipitation, air temperature, soil temperature and so on, could affect the NEP of grasslands through their influence on NPP and RH. At the seasonal scale, southern grasslands were strongly affected by the monsoon climate and characterized by high temperature and precipitation in wet seasons (Fig. 3b), but the NEP was much lower in dry seasons (Fig. 3b). The linear regression results showed that soil and air temperature accounted for most of the seasonal variation in NEP followed by precipitation and soil moisture (Table 1). We used stepwise regression to evaluate the influences of selected climatic variables on NEP and 71.4% (P < 0.001) of the seasonal variation in southern grasslands could be explained by precipitation and air temperature.

**Table 1:** Linear regressions between NEP (NEP(y), g C m^−2^ month^−1^) and climatic variables on monthly scale, covering radiation (NIRR, W m^−2^), soil temperature (Ts,  °C), mean air temperature (TAIR,  °C), mean soil moisture (SM, %) and precipitation (PRECIP, mm month-1) in southern China during 1961 to 2013.

Factors	Linear regressions	R^2^	P
NIRR	Y = 0.0291x−2.4353	0.25	0.099
Ts	Y = 0.4224x−5.9799	0.45	0.017
TAIR	Y = 0.3602x−5.6011	0.45	0.017
SM	Y = 1.3574x−40.153	0.34	0.047
PRECIP	Y = 0.0448x−7.4777	0.05	0.491

At the annual scale, there was a strong positive correlation between annual NEP and annual soil moisture, which meant that an increase in annual soil moisture would lead to an increase in the annual NEP of southern grasslands. However, statistical analysis indicated there was an insignificant correlation between annual NEP and annual mean air temperature or annual precipitation.

### Spatial Dynamics of Grassland NEP in Southern China

**Figure 4:**
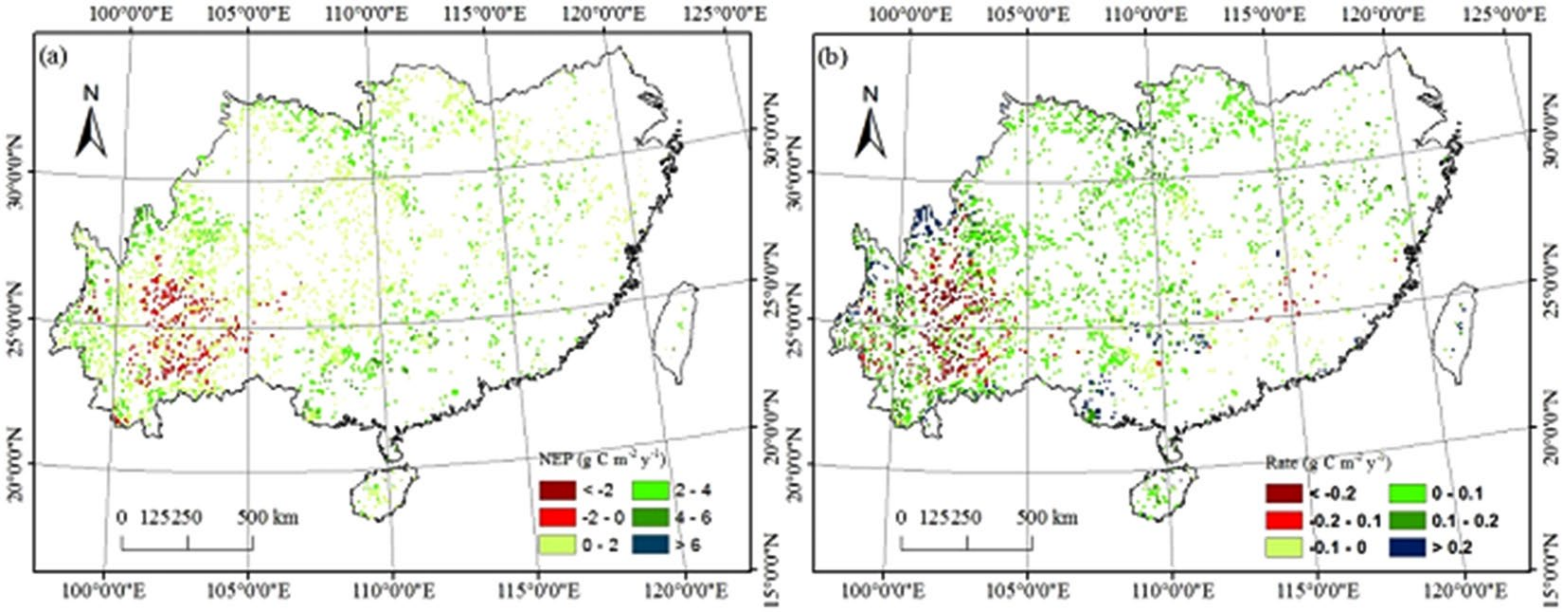
Spatial distribution patterns of average annual NEP (**a**) and annual NEP’s increasing rate (**b**) from 1961 to 2013 in southern grasslands (This picture was made by ArcGIS 10.2, http://esri.uconn.edu/).

From 1961 to 2013, TEM simulations indicated that southern grasslands acted as a carbon sink with a high standard deviation (3.38 Tg C y^−1^), which was the difference between the average annual NPP and RH of the total vegetated area of 0.29 × 10^6^ km^2^. Therefore, the annual NEP of southern grasslands in China was 1.53 g C m^−2^ y^−1^ with significant spatial variation; large carbon sinks were in the southern, western and northern portions of the southern grasslands (e.g., Guangxi, Hubei, and Sichuan Provinces), which experienced high precipitation and high temperatures. Central Yunnan Province, which has low temperatures and low precipitation mainly acted as a carbon source (Fig. 4a).

The spatial distribution of the areas with increasing NEP indicated that 71.81% of the study area tended to be a carbon sink from 1961 to 2013 (Fig. 4b). These areas are mainly in most areas in southern grasslands. Less than 22.26% of the total grasslands area acted as carbon sources. These areas were distributed mainly in the central of Yunnan province. Only approximately 5.93% of the study area was in a steady condition (0.00~0.01 g C m^−2^ y^−1^).

## Discussions

Only a few studies have been conducted on the carbon budget of southern grasslands (Table 2). For instance, Sun *et al*.^44^ estimated that southern grasslands sequestered carbon at a rate of 3.25 to 81.98 g C m^−2^ y^−1^ during 2001 to 2010 using an improved BIOME-BGC model. In contrast, we estimated a much lower carbon sink of 1.53 g C m^−2^ y^−1^ from 1961 to 2013, which was much lower than that of other types of grasslands, e.g., 158.00 g C m^−2^ y^−1^ for the temperate grasslands of northern China during the growing season^46^, 11.25 g C m^−2^ y^−1^ for the temperate grasslands of China^24^ by TEM from 1961 to 2007, 88.21 g C m^−2^ y^−1^ for the grasslands of Inner Mongolia^47^ and 4.00~192.50 g C m^−2^ y^−1^ for the grasslands of the Tibetan Plateau^20,48–52^. The estimates of Sun *et al*.^44^ were quite different from those of this study due to the large difference in the scopes of the two studies. The research by Sun *et al*.^44^ was based on samples from three typical grasslands (mountain steppe meadow, typical grassland mountain and slope and typical mountain meadow) in southern China, while this study covered all grasslands in southern China.

**Table 2:** Carbon budget of different study regions from different studies.

	Study area	Time	g C m-2 y-1	references
NEP	Southern grasslands	1961~2013	1.53	This study
		2001~2010	3.25~81.96	44
	Temperate grasslands in northern China	2000~2010	158.00	46
	Temperate grasslands in China	1961~2007	11.25	24
	Inner Mongolia grasslands	2001~2012	88.21	47
	Tibetan plateau grasslands	1961~2010	10.03	20
		1990s	28.00	48
		2002~2004	78.50~192.50	49
		—	55.26	50
		1981~2012	4.00	51
		2000~2001	55.47	52
NPP	Southern grasslands	1961~2013	318.15	This study
		2000~2011	471.62	39
		2010	389.14	40
		2001~2010	320.00	41
		1981~2000	1081.78	42
		2001~2010	656.30	43
		2001~2010	191.96~357.17	44
		1982~2012	356.79	45
	Inner Mongolia grasslands	2001~2012	278.83	47
		2001~2010	281.30	53
		1982~2002	290.23	54
		2002	259.90	55
		2002~2006	262.05	56
	Tibetan plateau grasslands	1961~2010	193.70	20
		1982~2009	120.80	19
		1990s	334.45	48
		1981~2002	199.00	51
		2001	161.12	57
		1982~1999	122.00	58
		2000~2001	92.50	52
		1980~1990	233.00	59
RH	Southern grasslands	1961~2013	317.16	This study
		1982~2012	314.46	45
		—	719.14	18
	Qinghai-Tibetan plateau grasslands	1990s	306.32	48
		2000~2001	79.61	52
	Temperate grasslands	1998~2000	390.00~866.00	60
		—	132.00~830.00	61
	Tropical grasslands	—	470.00~900.00	61

The TEM-simulated NPP of southern grasslands was 318.15 g C m^−2^ y^−1^, which was within the range of previous estimates^40,41,44,45^ of 191.96~389.14 g C m^−2^ y^−1^. In comparison, our estimated average regional NPP was lower than that simulation by Sun *et al*.^42^ (1081.78 g C m^−2^ y^−1^) using the Global Production Efficiency Model, by Sun *et al*.^43^ (656.30 g C m^−2^ y^−1^) using the improved comprehensive and sequential classification system (CSCS) and by Sun *et al*.^39^ (471.62 g C m^−2^ y^−1^) using a new climate productivity model. Our estimated annual NPP increased (0.88 g C m^−2^ y^−1^) from 1981 to 2000 and decreased (1.01 g C m^−2^ y^−1^) from 2000 to 2011.and Sun *et al*.^40^ similarly found an increasing trend from 1981 to 2000 with the GLO-PEM model and a decreasing trend from 2000 to 2011 by the improved CASA model. Earlier studies reported the NPP of different grasslands types. For instance, the simulated NPP ranged from 259.90 to 290.23 g C m^−2^ y^−1^ in Inner Mongolian grasslands^47,53–56^ and from 92.50 to 334.45 g C m^−2^ y^−1^ in Tibetan Plateau grasslands^19,20,48,51,52,57–59^. In general, the NPP of southern grasslands was similar to that of Inner Mongolian grasslands and slightly higher than the NPP of Qinghai-Tibetan Plateau grasslands per unit area.

Our simulated RH ranged from 300.90~339.00 g C m^−2^ y^−1^ from 1961 to 2013 with an annual average of 317.16 g C m^−2^ y^−1^, which was similar to that estimated by Liu^45^ with a range from 79.61 to 719.14 g C m^−2^ y^−1^ of the grasslands of the Qinghai-Tibetan Plateau^18,48,52^ and 132.00~866.00 g C m^−2^ y^−1^ for temperate grasslands^60,61^ but lower than 470.00~900.00 g C m^−2^ y^−1^ for tropical grasslands^60^.

The differences of in the grassland carbon budgets of these studies^37–45^ probably resulted for the following reasons. (1) The study regions and areas were different. The studies by Sun *et al*.^41^ and Liu^45^ were administered in south China and eastern Tibet, respectively, and the study by Sun *et al*.^38^ was administered in southern China, which had a grassland area of 0.6 × 10^6^ km^2^ of grasslands area. (2) The data sources in previous studies were also quite different. For example, Sun *et al*.^38^ and Liu^45^ used data from the moderate-resolution imaging spectroradiometer (MODIS) to estimate grassland NPP, and Sun *et al*.^41,44^ used observation data from 2009 to 2010 and data from the 1980 Chinese grassland resource inventory, respectively. (3) Different estimation methods were used. For example, Sun *et al*.^38^ estimated the NPP of grasslands based on the relationship between NPP and the normalized difference vegetation index (NDVI); Liu^45^ estimated NPP and carbon storage with the improved Comprehensive Sequence Classification System (CASA) model; Sun *et al*.^44^ estimated NPP and NEP based on the BIOME-BGC model and Sun *et al*.^42^ used the Global Production Efficiency Model to simulate NPP. (4) Different study periods were used. Liu^45^, Sun *et al*.^41,44^ performed their studies from 2001 to 2010; Sun *et al*.^42^ analyzed the temporal and spatial dynamics from 1981 to 2000; Sun *et al*.^38^ worked in 2011; and Sun *et al*.^39^ performed research from 2000 to 2011.

In this study, the area of the southern grasslands was approximately 0.29 × 10^6^ km^2^, with an annual mean NEP of 0.45 Tg C y^−1^, an annual mean NPP of 93.63 Tg C y^−1^ and an annual mean RH of 93.20 Tg C y^−1^. The annual mean NEP, NPP and RH of the grasslands of China were 8.44~54.40 Tg C^30,69^, 490.40~1392.00 Tg C^62–70^ and 320.00~3068.40 Tg C^71–73^, respectively, based on the results of previous studies (Table 3). Therefore, our results showed that southern grasslands accounted for 0.83~5.33% of the NEP, 6.73~19.09% of the NPP and 3.04~29.13% of the RH of the grasslands of China.

**Table 3:** Southern grasslands contribute to carbon budget in China’s grasslands.

	Time	(g C m^−2^ y^−1^)	Total (Tg C y^−1^)	Proportion (%)	references
NPP	2003	239.02	956.08	9.79	62
	1993~1999	271.00	1084.00	8.64	63
	1995	236.04	944.16	9.92	64
	1981~1998	348.00	1392.00	6.73	65
	1995	240.79	963.16	9.72	66
	2000	178.23	712.92	13.13	66
	—	275.00	1100.00	8.51	67
	1989~1993	298.65	1194.60	7.84	68
	1979~2008	279.4	1117.60	8.38	69
	2001	122.60	490.40	19.09	70
RH	—	767.10	3068.40	3.04	71
	—	282.33	1129.32	8.25	72
	—	80.00~350.00	320.00~1400.00	6.67~29.13	73
NEP	1979~2008	13.60	54.40	0.83	69
	1981~2000	2.11	8.44	5.33	30

The seasonal variation in NEP indicated that the dry season was a key period for annual net carbon emissions. In the wet season (June to August), southern grasslands acted as a weak carbon sink and were close to carbon neutral because the carbon fixed by the vegetation was equal to the amount released from the soil under warm and moist conditions. In contrast, the maximum monthly NEP was observed in May and October. Our study showed that the seasonal and annual variations in NEP were mainly driven by NPP.

Our analyses suggested that temperature (air temperature and soil temperature) was a key factor influencing the NEP of southern grasslands at the seasonal scale, accounting for 45% of the total variance in NEP. Future warming will tend to obviously positively correlate with the NEP of southern grasslands. Meanwhile, the results from our study suggested that water conditions (precipitation and soil moisture) also had strongly positive effects on the NEP of southern grasslands at the seasonal scale. Overall, all selected climatic variables could explain approximately 94.7% (P < 0.001) of the seasonal variation in NEP.

At the annual scale, the results indicated a positive relationship between the annual NEP and the annual soil moisture, but we found no remarkable relationship between annual NEP and any of other climatic variables. In contrast, Sun *et al*.^44^ found a negative correlation between annual NEP and annual mean temperature, but correlation analysis detected no significant relationship between precipitation and NEP in southern grasslands.

## Methods

### Study Region

**Figure 5:**
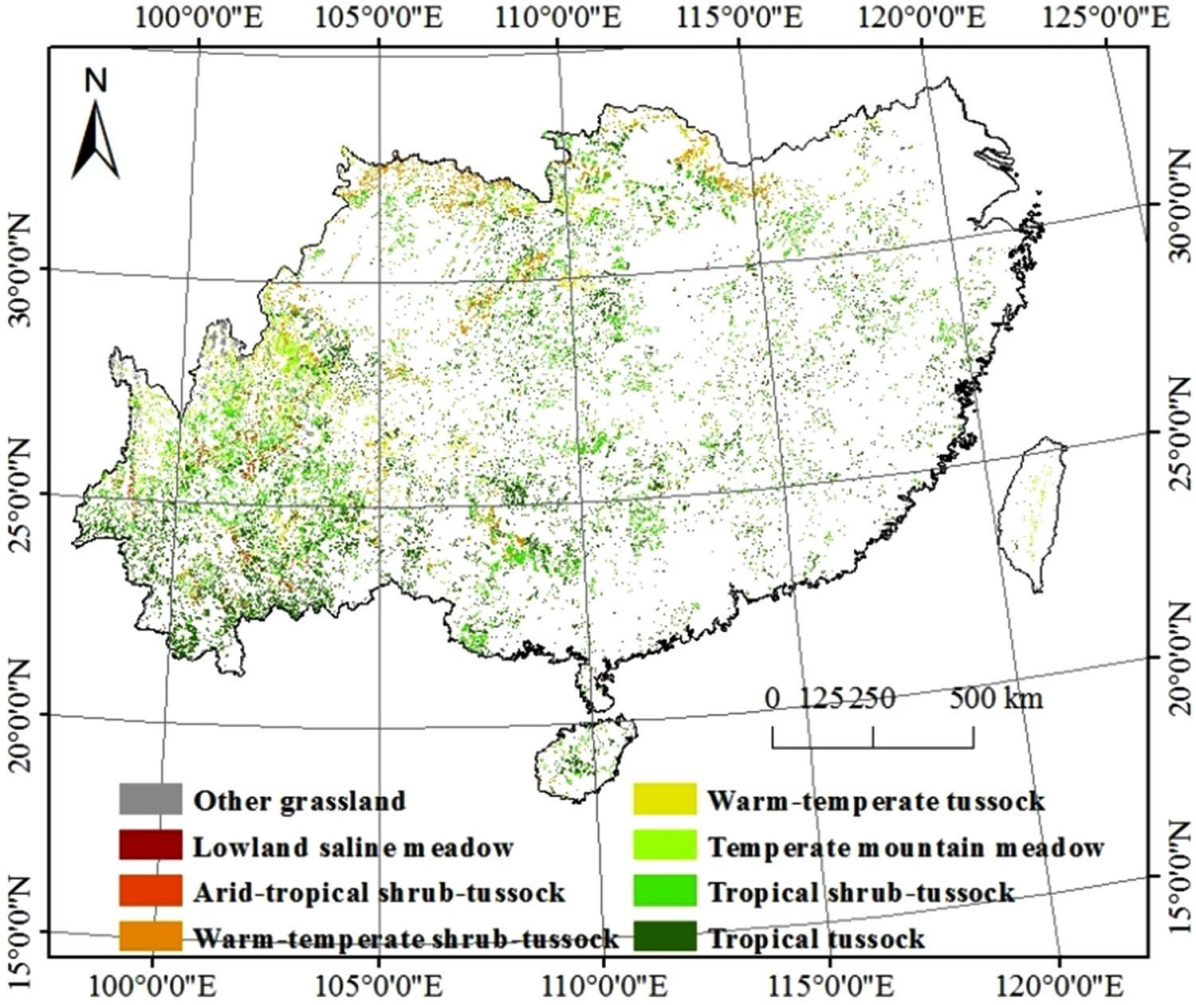
Distribution of southern grasslands (This picture was made by ArcGIS 10.2, http://esri.uconn.edu/).

The study region, which accounts for 24.60% of the total terrestrial area of China, covers approximately 2.36 × 10^6^ km^2^ and is located in southern China. Administratively, southern China includes 17 provinces and municipalities, e.g., Yunnan (excluding the Qinghai-Tibet Plateau), Sichuan (excluding the western portion of the Qinghai-Tibet Plateau), Guangxi, Hunan, and Hubei (Fig. 5). The southern grasslands cover an area of approximately 0.29 × 10^6^ km^2^, which accounts for 12.50% of southern China and 7.50% of the grasslands of China. The southern grasslands are mainly characterized by tropical tussock and tropical shrub-tussock vegetation based on the second grassland investigation in China, and the altitude varies by two or three staircases over complicated terrain. The eastern region is mainly composed of plains and hills, while the western region is mainly composed of basins and plateaus. Furthermore, the study area is mainly characterized by temperate monsoon, subtropical monsoon, and tropical monsoon climates. The summer is hot and humid and the winter is warm in the tropical monsoon climate region and cold in the subtropical monsoon climate region, although the air is relatively warm and moist^45^. The frost-free period is more than 300 d with abundant precipitation; the precipitation ranges from 800 mm to 1600 mm in most areas that are suitable for growing forage^74^.

### Data Source

The Terrestrial Ecosystem Model (TEM 5.0) requires input of monthly climate data sets (precipitation, temperature, cloud cover, and solar radiation) and longitude and latitude location information as well as soil texture, altitude, grassland distribution, and atmospheric CO_2_ data (Table 4). Daily climate data sets were obtained from basic and reference meteorological stations from National Meteorological Information Center, China Meteorological Administration (CMA) from 1961 to 2013. In this study, the TEM was applied to grid-cell for regional-scale simulation at a 1-month time scale, and each grid was 10 km × 10 km. First, we interpolated temperature and precipitation in southern China to a resolution of 10 km × 10 km using ANUSPLIN4.3^75^ and then calculated the solar radiation based on grid temperature and precipitation data sets using the method described by Thornton and Running^76^. Finally, we aggregated the daily climate data into monthly time steps to drive the TEM. The elevation data sets were taken from the National Fundamental Geographic Information System of China, and the soil texture data sets were collected from Nanjing Institute of Soil, the Chinese Academy of Sciences (CAS).The vegetation data sets were provided by the national grassland resource survey (Fig. 5). All the above spatial data sets were rearranged at a resolution of 10 km × 10 km. The atmospheric CO_2_ data from 1961 to 2013 were gathered from National Oceanic and Atmospheric Administration (NOAA), United States Department of Commerce (www.esrl.noaa.gov/gmd/ccgg/trends).

**Table 4:** Environmental Variables for the TEM.

Type	Data Name	Code	Unit	Time	Data Characteristics	Source
Location	Longitude	Lon	degree	—	constant	Longitude and latitude of meteorological stations
	Latitude	Lat	degree	—	constant	Longitude and latitude of meteorological stations
Altitude	Above sea surface	ELEV	m	—	constant	Altitude above sea surface of Meteorological Stations
Climate	Precipitation	PRECIP	mm	1961–2013	monthly	Meteorological data sharing network
	Temperature	TAIR	°C	1961–2013	monthly	Meteorological data sharing network
	Cloud Amount	CLDS	%	1981–2010	monthly	Meteorological data sharing network
	Radiation	NIRR	W m^-2^	1961–2013	monthly	Meteorological data sharing network
	CO2 Concentration		ppm	1961–2013	annual	NOAA published
Soil	Sand	PCTSAND	%	multi-year	constant	Second soil investigation
	Clay	PCTSILT	%	multi-year	constant	Second soil investigation
	Silt	PCTCLAY	%	multi-year	constant	Second soil investigation
Vegetation	Vegetation Type	TEMVEG	—	multi-year	constant	Grassland investigation

**Figure 6:**
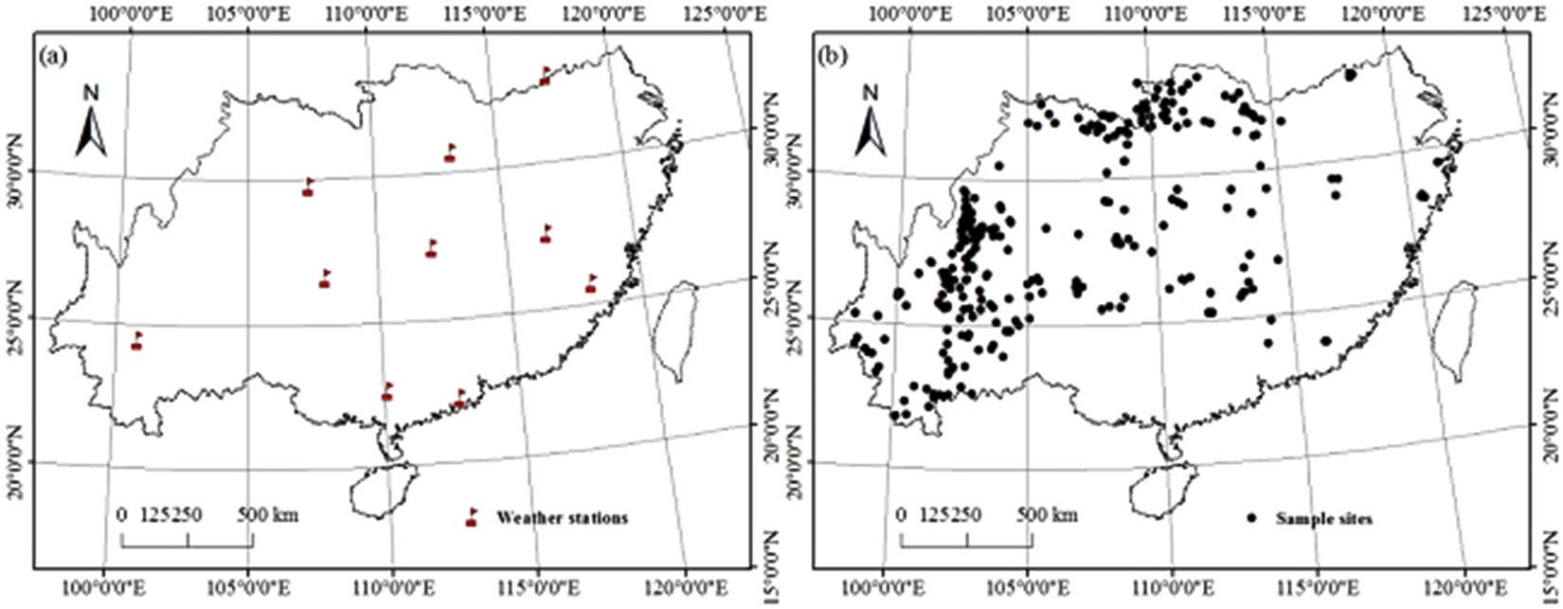
Distributions for 10 select meteorological stations in southern grasslands (**a**) and investigation stations to verified the model (**b**) (This picture was made by ArcGIS 10.2, http://esri.uconn.edu/).

The vegetation and soil carbon density observation data from 2011 to 2013 grasslands investigation were employed for TEM calibration and verification. As the southern grasslands are scattered, we randomly selected ten uniformly distributed meteorological stations in southern China to achieve the appropriate parameterization (Fig. 6a). Then, we chose survey samples within 50 km of each meteorological station using ArcGIS software as the calibration dataset (19 samples in total), and finally, the remaining survey samples were used to verify the TEM simulation results (Fig. 6b).

### TEM

The TEM is a regional- and global-scale, process-based biogeochemical ecosystem model that uses spatial climate, soil, vegetation and elevation data to simulate the sizes of carbon and nitrogen pools and the fluxes of terrestrial ecosystems at the month time scale^77,78^. The model framework and details have been described in previous studies^64,77–80^. This work used TEM version 5.0, which coupled TEM version 4.2 with a soil thermal model and is used widely for high altitudes and latitudes^78,81–85^.

At the monthly scale, the TEM defined the difference between net primary production (NPP) and soil heterotrophic respiration (RH) as net ecosystem production (NEP), and the difference between gross primary production (GPP) and plant autotrophic respiration (RA) was defined as NPP. These algorithms have been described in many previous studies^48,77,79,80^.

### Model Parameterization, Verification and Simulation

TEM parameterization requires monthly climate data sets and vegetation- and soil-specific parameters related to the nitrogen and carbon processes in vegetation and soil. For TEM calibration, the required parameters and their sources are shown in Table 5. The parameterization must follow three criteria within a certain tolerance (1%): (1) the simulated annual NPP and GPP are close to the measured values; (2) the simulated annual nitrogen uptake matches the measured value; and (3) the simulated annual NEP is close to zero^24,78^.

**Table 5:** Southern grassland ecosystem parameterization method.

Variables	Values	Sources and notes
CV	490	Average observational values from 2011 to 2013
NV	12	Estimated value
CS	1000	Average observational values from 2011 to 2013
NS	120	Estimate based on the soil C:N and Cs values in the southern regions
NAv	1.032	Estimate based on the average Nav:NS = 0.86%
GPP	370	Estimate based on GPP:NPP at approximately 1.5−2
NPP	185	See Sun *et al*.^37^
NPPSAT	277.5	Estimate based on NPPSAT:NPP at approximately 1.2−1.5
NUPTAKE	4.4	See Melillo *et al*.^86^ in Table 2

After TEM calibration, we used the soil and vegetation carbon density field observation data from the 2011 to 2013 grassland survey to verify the simulated results by comparing the average simulated results for 2011~2013 with the observations. When multiple sample sites located in one grid cell, we calculated the mean value of these sample sites.

We then extrapolated the parameterized and verified TEM to southern China for 1961 to 2013. For zone modeling, we first ran the TEM at each grid cell to equilibrium using the long-term average monthly meteorological data sets of 1961~2013. Then, to explain the impact of inter-annual climate variability, we used the 1961~1975 climate data to spin up 45 years of the model of the undisturbed ecosystem. Finally, we used atmospheric CO_2_ concentrations and monthly climate data for 1961~2013 to run the model.

Lastly, after model simulation, we aggregated the simulated results from each grid to the whole study region by multiplying the grasslands area data and the simulated results grid by grid.

### Data availability statement

The datasets used during the current study such as vegetation and soil carbon density are available from grasslands investigation during 2011 to 2013 (this project was funded by the Strategic Priority Research Program—Climate Change: Carbon Budget and Related Issues of the Chinese Academy of Sciences [grant number XDA05050408]), climate data sets are available from National Meteorological Information Center, China Meteorological Administration (CMA), the elevation data sets are available from the National Fundamental Geographic Information System of China, the soil texture data sets are available from Nanjing Institute of Soil, the Chinese Academy of Sciences (CAS), the vegetation data sets are available from the national grassland resource survey, and the atmospheric CO_2_ data is availabe from National Oceanic and Atmospheric Administration (NOAA), United States Department of Commerce (www.esrl.noaa.gov/gmd/ccgg/trends).
